# The Usefulness of Indirect Immunofluorescence in Pemphigus and the Natural History of Patients With Initial False-Positive Results: A Retrospective Cohort Study

**DOI:** 10.3389/fmed.2018.00266

**Published:** 2018-10-17

**Authors:** Khalaf Kridin, Reuven Bergman

**Affiliations:** ^1^Department of Dermatology, Rambam Health Care Campus, Haifa, Israel; ^2^Rappaport Faculty of Medicine, Technion-Israel Institute of Technology, Haifa, Israel

**Keywords:** pemphigus, indirect immune fluorescence assay, monkey esophagus, pemphigus vulgaris, pemphigus foliaceus, false positive, sensitivity, specificity

## Abstract

The specificity and the predictive values of indirect immunofluorescence (IIF) in real-life settings is yet to be firmly established. The natural history of patients with false-positive results has not been sufficiently elucidated. The primary aim of the current study is to evaluate the diagnostic value of IIF analysis on monkey esophagus in pemphigus, utilizing a large cohort arising from the real-life experience of a tertiary referral center. The secondary endpoint was to determine the clinical outcomes of patients with false-positive results. This was a retrospective cohort study including all patients who were tested for the presence of intercellular autoantibodies by IIF on monkey esophagus between 2000 and 2017. Overall, 770 sera from different individuals were tested by IIF microscopy. Of those, 176 patients had been diagnosed with pemphigus vulgaris (PV) and 29 patients with pemphigus foliaceus (PF). The sensitivity of this immunoassay was significantly higher for the diagnosis of PV (87.4%; 95% CI, 81.5–91.9%) as compared to PF (69.0%; 95% CI, 49.2–84.7%; *P* = 0.018). The specificity for the diagnosis of pemphigus was 93.5% (95% CI, 91.1–95.4%). Patients with false-positive results (*n* = 37) were followed for a median duration of 5.3 years contributing 280.8 person-years. Thirty patients (81.1%) were eventually diagnosed clinically and immunopathologically with subepidermal autoimmune bullous diseases, whereas the remaining patients (18.9%) were diagnosed clinically and histologically with other inflammatory dermatoses, but none of them developed pemphigus during the follow-up duration. Of note, 7.0% (*n* = 23) of all patients diagnosed with bullous pemphigoid (BP) in the same period (*n* = 328) were tested positive for IgG intercellular antibodies. Histopathological review of the biopsy specimens of these patients did not reveal acantholysis. In conclusion, the predictive value of negative test in IIF on monkey esophagus is particularly reliable to exclude a diagnosis of pemphigus. Individuals tested positive for intercellular antibodies without an initial overt pemphigus did not show an increased risk for developing pemphigus subsequently. A sizable fraction of patients with BP showed circulating intercellular autoantibodies by IIF, without a histopathological evidence for acantholysis.

## Introduction

Pemphigus is a rare, chronic, potentially life-threatening, autoimmune bullous disease of the skin and the mucous membranes. The two major subtypes of pemphigus are pemphigus vulgaris (PV) and pemphigus foliaceus (PF). The etiopathogenesis underlying the development of the disease is characterized by acantholysis and intraepidermal blister formation, resulting from IgG autoantibodies directed against desmoglein (Dsg) 3 (PV) and/or Dsg 1 (PF), two transmembrane desmosomal glycoproteins (1, 2).

An essential element of the diagnosis of pemphigus is the detection of circulating intercellular antibodies. Despite the detailed knowledge of pemphigus antigens and the development of increasing number of sensitive and specific assays for the detection of circulating autoantibodies, including Western blotting of cell-derived and recombinant forms of the target antigens, immunoprecipitation, and enzyme-linked immunosorbent assay (ELISA), the diagnosis of pemphigus in most laboratories still relies chiefly upon indirect—immunofluorescence (IIF) technique. Although a universally sensitive substrate has not been established, IIF on monkey esophagus has been elucidated as the most sensitive screening test (3–5). The value of IIF titers in disease monitoring has been a subject of debate, with conflicting results throughout the years. Although early studies suggested that intercellular antibodies levels measured by IIF were a useful marker of disease activity (6–8), later studies concluded that IIF titers did not always correlate with the disease severity, and are not consistent enough to serve as a guide for therapy or for monitoring the disease activity (9–12).

IIF is positive in approximately 70–90% of pemphigus patients but lacks the ability to differentiate definitively between PV and PF since both have IgG antibodies directed against keratinocyte cell surface (4, 13, 14). The sensitivity of this assay varies according to the specificity of the epithelial substrate the sera are incubated with (4, 14–17). To the best of our knowledge, the specificity and the predictive values of this immunological assay was not estimated in real-life settings in the past. Unlike controlled trials examining the diagnostic features of this immunoassay under optimal setting in the absence of confounding factors, real life studies inform on the effectiveness of an analysis when performed in routine circumstances, by including all patients with initial suspicion of autoimmune bullous disease and not only patients and healthy control subjects. Furthermore, the natural history of patients with false-positive results is yet to be fully elucidated.

The aim of the current study is to evaluate the sensitivity, specificity and predictive values of IIF analysis on monkey esophagus utilizing a large cohort extracted from the real-life experience of a tertiary center throughout an extended duration. Our secondary endpoint was to determine the clinical outcomes and histological features of patients tested positive for intercellular antibodies without an initial diagnosis of pemphigus (false-positive patients).

## Methods

This was a retrospective cohort study that included all patients who were tested for the presence of intercellular autoantibodies by (IIF) in Rambam Health Care Campus, Haifa, Israel, throughout the years 2000–2017. The current study was approved by the institutional ethical board of our medical center.

### Patients and case definition

The clinical and immunopathological criteria for the diagnosis of PV were: (1) presence of skin blisters and/or erosions on mucous membranes; (2) suprabasal intraepidermal acantholysis on histopathological examination of skin and/or mucosa; and (3) intraepidermal intercellular IgG and/or C3 deposits by direct immunofluorescence (DIF); or intercellular circulating antibodies demonstrated by using monkey esophagus and a standard IIF technique; or the presence of anti-Dsg 3 ± anti-Dsg 1 autoantibodies, measured by ELISA (UROIMMUN Medizinische Labordiagnostika AG; Lübeck) or immunoblotting (on human dermal and epidermal extracts as antigenic substrate) (18).

The clinical and immunopathological criteria for the diagnosis of PF were: (1) presence of skin blisters or erosions; (2) lack of mucosal lesions; (3) intraepidermal acantholysis compatible with PF on histopathological examination; and (4) intraepidermal intercellular IgG and/or C3 deposits by DIF; or intercellular circulating antibodies demonstrated by a standard IIF technique; or presence of anti-Dsg 1 autoantibodies, with lack of anti-Dsg 3 autoantibodies, measured by ELISA or immunoblotting (18).

The differentiation between the different phenotypes of PV (isolated mucosal, isolated cutaneous, or mucocutaneous) was grounded on the clinical and endoscopic (in cases of laryngeal involvement) presentation at the onset of the disease, without referring to serological analyses to ascertain that those with the isolated mucosal disease had only anti-Dsg 3 antibodies, and those with mucocutaneous had both anti-Dsg 3 and anti-Dsg 1 antibodies.

### Indirect immunofluorescence

All sera were tested in serial dilution for intercellular antibodies by IIF. IIF assay using monkey esophagus as the substrate was performed according to a standard technique (18). Sera samples were incubated with monkey esophagus, and fluorescein-labeled goat anti-human IgG sera (Tago, Camarillo, CA) were added subsequently. Each serum sample was examined under fluorescence microscopy. Serum samples were considered to be positive if they stained epidermal intercellular spaces at a titer of ≥20.

### Histopathology

Hematoxylin-eosin stained cutaneous biopsies of patients with false-positive results were re-examined for evidence of acantholysis.

### Statistical analysis

All continuous parameters were expressed as mean values ± standard deviation (SD). Categorical variables were expressed as proportions. Comparisons of percentages between different patient groups were carried out using the Chi-square test. To determine the sensitivity and specificity of the IIF immunoassay, receiver operating curves (ROC) were analyzed. For sample proportions, 95% confidence interval (CI) was computed using the Clopper–Pearson exact binomial proportion interval method as fitting. CIs for the likelihood ratios were calculated using the “Log method.” CIs for the predictive values were the standard logit CIs given by Mercaldo et al. (19). Figures with *P*-values < 0.05 were considered statistically significant. Statistical analysis was performed using IBM SPSS statistics software, version 23 (IBM Corp, Armonk, New York, USA) and MedCalc Statistical Software (version 16.4.3, MedCalc Software, Ostend, Belgium).

## Results

### Clinical and demographic characteristics of the study participants

Overall, 770 sera from different individuals were tested for the presence of intercellular autoantibodies by IIF microscopy between the years 2000 and 2017. Of those, 176 patients had been diagnosed with PV and 29 patients with PF. IIF analysis was performed on the sera of 174 (98.9%) patients diagnosed with PV and all patients with PF before the initiation of any immunosuppressive therapy. With regard to patients with PV, the most frequent clinical phenotype was mucocutaneous (*n* = 106; 60.2%), followed by isolated mucosal disease (*n* = 63; 35.8%) and isolated cutaneous disease (*n* = 7; 4.0%). The demographic and clinical characteristics of the patients with pemphigus included in the analysis are demonstrated in Table 1.

**Table 1:** Demographic and clinical characteristics of patients with pemphigus whose sera were tested by indirect immunofluorescence microscopy.

	**Pemphigus vulgaris (*n* = 174)**	**Pemphigus foliaceus (*n* = 29)**
**AGE AT DIAGNOSIS**		
Mean ± SD	55.5 ± 15.3	57.1 ± 21.4
Median (range)	55 (20–90)	63 (20–87)
% female (n)	65.9% (116)	34.5% (10)
**CLINICAL PHENOTYPE, % (N)**		
Isolated mucosal	35.6 (62)	NA
Mucocutaneous	60.4 (105)	NA
Isolated cutaneous	4.0 (7)	NA

*n, number; SD, standard deviation*.

### The diagnostic value of IIF on monkey esophagus

Of the 174 sera from PV patients tested by IIF, 152 were positive for intercellular antibodies against monkey esophagus, corresponding to a sensitivity of 87.4% (95% CI, 81.5–91.9%; Table 2). When PV patients were divided according to the clinical phenotype, IIF sensitivity was comparable for those with isolated mucosal (87.1%; 95% CI, 76.2–94.3%) and mucocutaneous phenotypes (87.6%; 95% CI, 79.8-93.2%; *P* = 0.925). Patients with isolated cutaneous disease had lower sensitivity (57.1%; 95% CI, 18.4–90.1%), but the small size of this subgroup (*n* = 7) hinders drawing meaningful comparisons. The sensitivity of IIF on monkey esophagus in PF patients was only 69.0% (95% CI, 49.2–84.7%; Table 2). Taken together, the sensitivity of this immunoassay was significantly higher for the diagnosis of PV as compared to that of PF (*P* = 0.018).

**Table 2:** Evaluation of indirect immunofluorescence immunoassay on monkey esophagus.

	**Pemphigus vulgaris**		**Pemphigus foliaceus**	
	**Value (%)**	**95% confidence interval**	**Value (%)**	**95% confidence interval**
Sensitivity	87.1	76.2–94.3%	69.0	49.2–84.7%
Positive predictive value	80.4	75.0–84.9%	35.1	26.7–44.5%
Negative predictive value	96.0	94.2–97.3%	98.3	97.2–99.0%
Positive likelihood ratio	13.4	9.8–18.4	10.6	7.1–15.7
Negative likelihood ratio	0.14	0.1–0.2	0.33	0.2–0.6

Overall, 770 patients were tested by IIF microscopy, including 567 (73.6%) patients who did not have an established diagnosis of pemphigus. Of those, 37 (6.5%) were tested positive for intercellular antibodies by IIF. Altogether, the specificity of this immunoassay for the diagnosis of pemphigus was as high as 93.5% (95% CI, 91.1–95.4%).

The positive predictive value (PPV) of IIF on monkey esophagus for the diagnosis of PV was 80.4% (95% CI, 75.0–84.9%) and the negative predictive value (NPV) was 96.0% (95% CI, 94.2–97.3%). The positive likelihood ratio (PLR) was 13.4 (95% CI, 9.8–18.4), while the negative likelihood ratio (NLR) was 0.14 (95% CI, 0.1–0.2). With regard to PF, the PPV of this assay was 35.1% (95% CI, 26.7–44.5%), whereas the NPV was 98.3% (95% CI, 97.2–99.0%). PLR and NLR were 10.6 (95% CI, 7.1–15.7) and 0.33 (95% CI, 0.2–0.6), respectively (Table 2).

### Characterization of false-positive patients

The clinical features of the 37 non-pemphigus patients tested positive for intercellular antibodies were analyzed. These patients were followed for a median duration of 5.3 years (range, 0.7–16.9 years), contributing 280.8 person-years.

**Figure 1:**
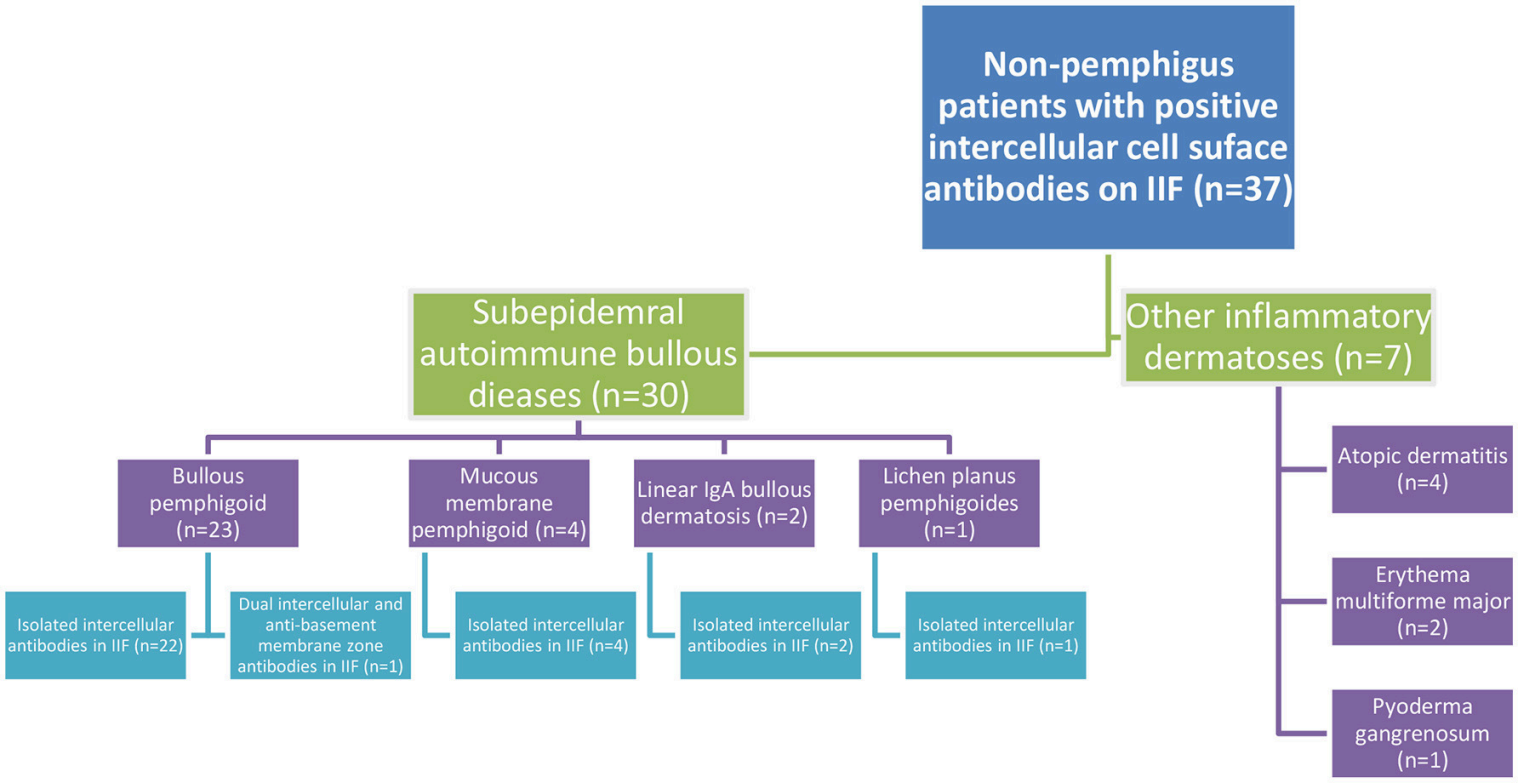
Flowchart of the clinical characteristics of non-pemphigus patients tested positive for intercellular cell surface antibodies in IIF on monkey esophagus.

Thirty patients (81.1%) were eventually diagnosed clinically and immunopathologically with subepidermal autoimmune bullous diseases (SAIBD); 23 patients (62.2%) with bullous pemphigoid (BP), of whom one patient had coexisting psoriasis, 4 patients (10.8%) with mucous membrane pemphigoid, 2 patients (5.4%) with linear IgA bullous dermatosis (had IgA intercellular antibodies), and one patient (2.7%) with lichen planus pemphigoides. Apart from one patient with BP having dual intercellular and anti-basement membrane zone (BMZ) antibodies, all the remaining 29 (96.7%) patients with SAIBD had isolated intercellular antibodies detected by IIF. The diagnosis of theses SAIBD was grounded on suggestive clinical presentation, compatible histopathology, and linear deposits of immunoreactants along the BMZ by DIF and/or the presence of circulating IgG antibodies against the immunodominant domain of BP180 (NC-16A) using ELISA (in cases of BP) (20). Intercellular tissue-bound antibodies was not detected in any of these patients, as DIF microscopy revealed isolated linear deposition along the BMZ in all 30 cases. Altogether, 7.0% (*n* = 23) of all patients diagnosed with (BP) in the same period (*n* = 328) were tested positive for IgG intercellular antibodies by IIF on monkey esophagus (Figure 1).

The remaining 7 patients were diagnosed clinically and histopathologically with various inflammatory dermatoses; 4 patients with atopic dermatitis, 2 patients with erythema multiforme major, and one patient with pyoderma gangrenosum (Figure 1).

None of the above 37 seropositive patients have subsequently developed pemphigus throughout the duration of follow up. Lesional biopsy specimens of 32 patients with the false-positive results were reviewed, and no histopathological sign of acantholysis was detected.

## Discussion

The results of the current study indicate that IIF microscopy on monkey esophagus is a more sensitive test for the detection of circulating intercellular antibodies in PV than in PF. The specificity of the assay is high when used in routine practice, and the predictive value of a negative test is particularly reliable to exclude the diagnosis of pemphigus. Individuals tested positive for intercellular antibodies by IIF, without initial overt pemphigus, did not show an increased risk for developing pemphigus subsequently. None of the false-positive patients had histological evidence of acantholysis.

The positivity rate of IIF in previous cohorts varied depending on the epithelial substrate which the sera were incubated with. In a recent German study, intercellular epithelial staining on monkey esophagus by IIF was observed in 100.0 and 98.0% of 65 patients with PV and 50 patients with PF, respectively (5). In an earlier British study, the diagnostic sensitivity of IIF on monkey esophagus was 100.0% in 20 patients with PV and 67.0% in 9 patients with PF (14). Jiao and Bysryn (4), examining 41 sera from PV patients and 22 sera from PF patients, had demonstrated that 87.0 and 86.0% of patients with PV and PF, respectively, were tested positive for intercellular antibodies by IIF when the assays were conducted on 2 different substrates simultaneously (monkey and guinea pig esophagus). In a small-scale study from Singapore, Ng PPL et al. (15) reported that all 13 PV patients and 11 of 12 PF patients had positive IIF on monkey esophagus resulting in sensitivities of 100.0 and 91.7%, respectively. In two previous serological studies performed in our center, the sensitivity of IIF on monkey esophagus was estimated at 96.0 and 81.0% for the diagnosis of PV in small cohorts of 25 and 32 patients, respectively (18, 21). Our study examined a 2- to 10-fold larger number of patients and found that the sensitivity for PV (87.4%) was lower than reported in most studies, whereas the sensitivity for PF (69.0%) was within the range of previous studies (4, 14). The predictive value of a negative test in our study (96.0%) was found to be particularly reliable to rule out a diagnosis of pemphigus. The large sample size in our study provides sufficient statistical power to exclude chance as the basis for the findings and sustains its external validity.

Several studies have estimated the the specificity of IIF by testing—healthy control subjects. A recent German study which examined 115 pemphigus patients estimated the specificity at 89.1% (5). Another study comprising 33 Chinese pemphigus patients depicted that the specificity of this immunoassay was 91.8% (22). A similar specificity (94.8%) was reported in a previous small-scale (*n* = 25) study from our center (18). A higher specificity of 100.0% was demonstrated by another serological study including 32 Israeli PV patients (21). Apart from the latter, the specificity revealed in our study (93.5%) is comparable with that reported in other studies. It is noteworthy that all the aforementioned studies enrolled healthy control subjects in order to identify the specificity rates, most of them characterized by low pretest probability. Conversely, our study was undertaken in a real-life setting and consisted of individuals whose clinical presentation raised suspicion for autoimmune bullous diseases. Thus, the pretest probability was moderate to high in most control subjects. Our findings, therefore, should represent the real diagnostic value of this immunoassay in the everyday clinical practice more efficiently than studies recruiting healthy participants.

### Interpretation of findings

The higher sensitivity of this immunoassay for the diagnosis of PV is conceivable in light of the fact that monkey esophagus is a mucosal substrate with high expression of Dsg3, the main autoantigen in PV (4, 9, 14). This substrate is less sensitive for PF patients with circulating anti-Dsg1 autoantibodies due to lower expression of Dsg1. IIF positivity depends on both the quantities of anti-Dsg1 and anti-Dsg3 antibodies in the test serum and the relative expression of Dsg1 and Dsg3 in the epithelial substrate. It was demonstrated that Dsg1-rich epithelial substrates like guinea pig esophagus and human skin were more sensitive than monkey esophagus for the diagnosis of PF (3, 4). One study showed that the sensitivity of IIF on human skin was greater than on monkey esophagus in patients with PF, whereas the sensitivity of IIF on monkey esophagus was higher than on human skin in patients with PV (14). Thus, some authors claim that the combination of a Dsg1-rich substrate, such as guinea pig esophagus or human skin, and a Dsg3-rich substrate, such as monkey esophagus, is crucial prerequisite to increase the sensitivity of IIF when screening the sera of pemphigus patients. It is noteworthy that other authors reported conflicting results suggesting that the sensitivity of IIF on monkey esophagus was comparable in PV and PF patients (5, 15). The reason for this discrepancy is unknown.

The predominance of SAIBDs among patients with false-positive assays may be ascribed to the “epitope spreading phenomenon”; a process in which a primary autoimmune or inflammatory cutaneous process may induce structural alterations in epidermal antigens (23, 24). The immune responses can spread over the disease course and recognize epitopes which are different from the original target. If it occurs in the same molecule, it is termed “intramolecular epitope spreading” (25), and if this immunological response involve epitopes on other proteins, it is then termed “intermolecular epitope spreading” (26). Regarding the relatively high false-positivity among patients with SAIBDs, it may postulated that intercellular cell-surface antigens that were previously concealed from the immune system became exposed, leading to the induction of a secondary autoimmune response that may be reflected by the production of non-pathogenic intercellular autoantibodies (24). A remarkable multicenter longitudinal study examined the profile of IgG autoantibody response to distinct BP180 and BP230 epitopes during the clinical course of 35 BP patients (27). Epitope spreading events were detected in up to 50% of cases, mainly intramolecular epitope spreading events consisting of early IgG reactivity with extracellular epitopes, which was followed by IgG reactivity with intracellular epitopes of BP180. This study did not investigate the development of intermolecular epitope spreading against Dsg1/3 (27).

Sami et al. (28) presented 13 patients with an initial immunopathological diagnosis of BP who subsequently demonstrated coexistent serological features of both BP and PV and failed to respond to conventional systemic therapy. IIF using monkey esophagus as substrate revealed high levels of intercellular cell surface antibodies in all patients (7 in conjunction with anti-BMZ antibodies and 6 without anti-BMZ antibodies). Additionally, all 13 patients had anti-Dsg3 antibodies and 9 had anti-Dsg1 antibodies on ELISA. The administration of intravenous immunoglobulin (IVIg) resulted in effective clinical response and the maintenance of prolonged clinical remission. In view of their findings, the authors recommended performing detailed serological re-evaluation and considering a dual diagnosis of BP and PV in patients with an initial diagnosis of BP who are nonresponsive to conventional therapy. Unlike these findings, the great majority of our “false-positive” BP patients had isolated intercellular antibodies without simultaneous detection of anti-BMZ antibodies by IIF. While Sami et al. (28) attributed a pathogenic role for intercellular antibodies detected in patients with BP which supposedly rendered them more recalcitrant for conventional therapy, we did not find any distinct clinical features for BP patients with intercellular antibodies. In addition, histological review of lesional biopsy specimens did not reveal acantholysis, thus arguing against a pathogenetic role for intercellular antibodies detected in IIF in this subgroup.

In 2001, Sami and Ahmed (29) reviewed the literature and summarized 17 reported patients with mutual features of both BP and PV. Of whom, 83% had serum antibodies typical of PV. The present study demonstrated that this phenomenon occurred in 7% of BP patients. A notable case of 26-years old woman presenting with tissue-bound and circulating antibodies suggestive of both pemphigoid gestationis (PG) and pemphigus in DIF and IIF, respectively, was reported (30). Clinically, she presented with erythematous eruption on the lower abdomen and thighs the day following delivery. The patient behaved clinically as a typical case of PG exhibiting a good response to moderate dose of oral corticosteroids, and lacked conventional clinical manifestation of pemphigus (30).

It was evidenced that IgG4 is the major subclass of autoantibodies in active pemphigus (31–33). Dsg-specific autoantibodies in pemphigus patients with active disease tend to preferentially associate with IgG4 subclass (33, 34). In both PV and PF, patients with active disease demonstrate Dsg-reactive IgG4 and IgG1, while patients in remission and some healthy relatives of patients with pemphigus can demonstrate only anti-Dsg IgG1 (34–36). A recent study had revealed that serum IgG4, but not other IgG subclasses, was enriched in patients with pemphigus compared with unaffected individuals (32). Additionally, IgG4 depletion in PV sera diminished pathogenicity in a keratinocyte dissociation assay and depicted that affinity-purified IgG4 is more pathogenic than other serum IgG fractions (32). Another study found IgG4 to be the exclusive subclass that differentiates PV patient subgroups based on different disease morphologies and disease durations (33). Moreover, an IgG4-specific Dsg ELISA was verified to have greater sensitivity and specificity than a total IgG Dsg ELISA in identifying active disease in endemic PF, suggesting a more substantial clinical association of pathogenic antibodies with IgG4 rather than with other IgG subclasses (37). It is of great interest to explore whether false-positive patients had IgG4 anti-cell surface antibodies or, alternatively, IgG2 and IgG3 autoantibodies which have not been associated with a pathogenic role in pemphigus (38, 39). Given the retrospective data collection and the unavailability of the sera, characterization of the specific subclass of IgG antibodies could not be performed.

### Strengths and limitations

The sample size is large, and all the analyses were performed before the initiation of any immunosuppressive medications which could interfere with the results. Our study has some limitations to consider. First, the phenotypes of PV patients were not categorized according to the immunoserological profile. Second, although the immunoassays were performed in the same laboratory using the same substrate, at least 2 technicians analyzed the results of this subjective technique. The titers and the specific subclass of autoantibodies, as well as the specific pattern of deposition were not evaluated systematically in all patients. Thus, we could not investigate the association between autoantibodies levels and subclasses and the clinical characteristics.

It is noteworthy that a growing body of evidence accumulated in the last decade to signify the high sensitivity of Dsg 1 and Dsg 3-ELISA (40, 41). Many authors recommend utilizing this technique, available as mono-analyte or multi-analyte systems, as an easier technique. However, IIF is still widely used for the immunoserological diagnosis of pemphigus, specifically in low-income countries.

In conclusion, IIF microscopy on monkey esophagus is a more sensitive immunoassay for the detection of circulating intercellular autoantibodies in PV than in PF. The specificity of the assay is relatively high when used in real-life clinical settings, and the negative predictive value is particularly reliable to exclude the diagnosis of pemphigus. A notable proportion of patients with BP (7.0%) showed false-positive circulating intercellular autoantibodies by IIF. None of the patients with false-positive results demonstrated a histological evidence for acantholysis or developed pemphigus during the follow-up duration, which argues against a pathogenic role of intercellular antibodies in this subgroup.

## Ethics statement

This retrospective, non-interventional study was approved by the institutional ethical board of Rambam Health Care Campus, waiving patient written informed consent.

## Author contributions

KK substantial contributions to the conception or design of the work; or the acquisition, analysis, or interpretation of data for the work. KK and RB drafting the work or revising it critically for important intellectual content. KK and RB final approval of the version to be published.

### Conflict of interest statement

The authors declare that the research was conducted in the absence of any commercial or financial relationships that could be construed as a potential conflict of interest.
